# 778. Prediction of Bloodstream Infection Events and Infections of the Lower Respiratory Tract in ICU Patients: Expected and Unexpected Infections

**DOI:** 10.1093/ofid/ofab466.975

**Published:** 2021-12-04

**Authors:** Rogério Pereira, Débora da Silva, Sinval Silva, Anna Sophia F Gonçalves, Carolina Ranyere, Daniel Fontes, Pedro Duarte, Lourenço Ferreira, Rodrigo Santos, Andreza De Freitas, Thaís Guimarães, Leonardo De Faria, Bráulio R G M Couto

**Affiliations:** 1 Hospital Felício Rocho, Belo Horizonte, Minas Gerais, Brazil; 2 Centro Universitário de Belo Horizonte - UniBH, Belo Horzonte, Minas Gerais, Brazil

## Abstract

**Background:**

Bloodstream infection (BSI) - Central and Non-Central Line Associated - and infections of the lower respiratory tract (RESP) - pneumonia and non pneumonia lower respiratory infections - are some of the main causes of unexpected death in Intensive Care Units (ICUs). Although the leading causes of these infections are already known, risk prediction models can be used to identify unexpected cases. This study aims to investigate whether or not it is possible to build multivariate models to predict BSI and RESP events.

**Methods:**

Univariate and multivariate analysis using multiple logistic regression models were built to predict BSI and RESP events. ROC curve analysis was used to validate each model. Independent variables: 29 quantitative parameters and 131 categorical variables. BSI and RESP were identified using Brazilian Health Regulatory Agency protocols with data collected between January and November 2020 from a medical-surgical ICU in a Brazilian Hospital. Definitions: if an infection is 5% or less likely to occur according to the model used and it eventually occurs, it will be classified as “unexpected”, or else, if an infection is 10% or less likely to occur, it will be classified as “probably unexpected”. Otherwise, infections will be classified as “expected”. Patients with a 30% or more risk for BSI or RESP will be classified as “high risk”.

**Results:**

A total of 1,171 patients were accessed: 70 patients with BSI (95% confidence interval [CI], 3.1%-5%), 66 patients with RESP (95% CI, 2.9%-4.7%), 235 deaths (95% CI, 11.8%-14.9%). Of the 160 potential risk factors evaluated, logistic models for BSI and RESP identified respectively five and seven predictors (Tables 1 and 2, and Figure 1). Patients admitted to the ICU with Covid-19 had a three fold BSI risk and five times more RESP risk than patients without this diagnosis.

Table 1. Independent predictors of Infections of the Lower Respiratory Tract in ICU: results of multivariate analysis performed using a logistic regression model.

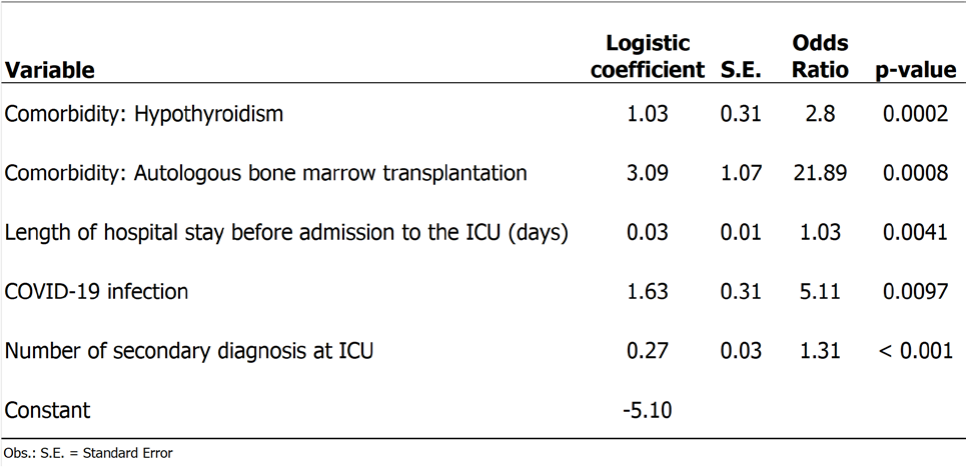

Table 2. Independent predictors of Bloodstream Infection Events in ICU (Central Line-Associated BSI + Non-central Line Associated BSI): results of multivariate analysis performed using a logistic regression model.

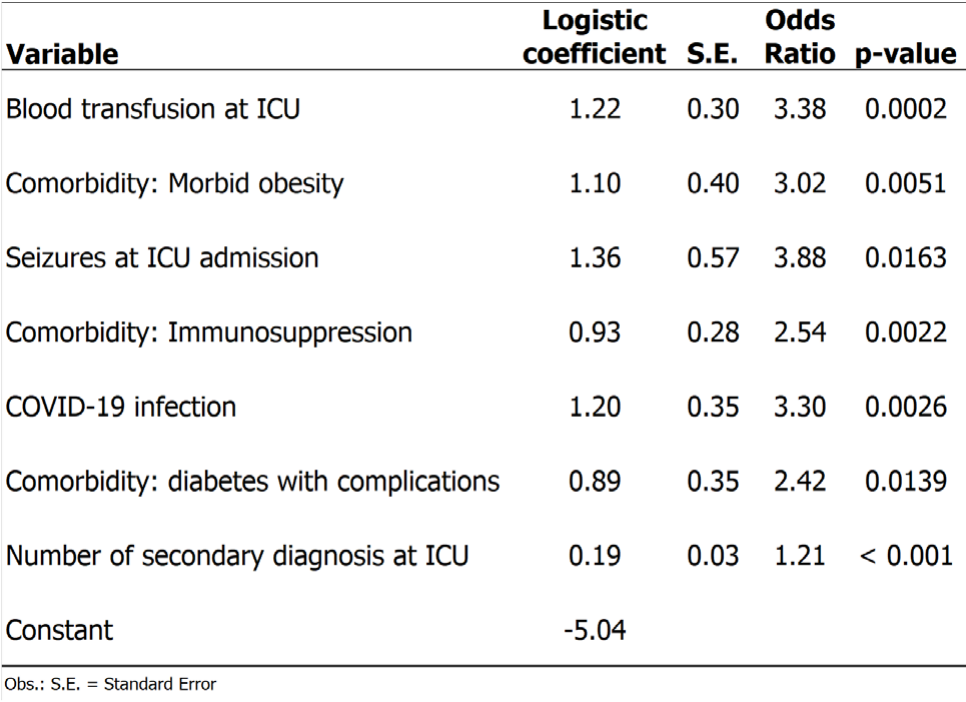

Figure 1. Receiver operating characteristic (ROC) curve for the fitted models: area under the ROC Curves were higher than 0.85 for both models.

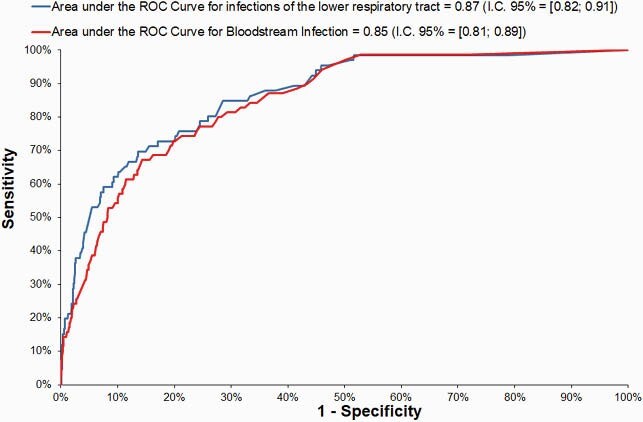

**Conclusion:**

The built models make possible the identification of the expected infections and the unexpected ones. Three main course of actions can be taken using these models and associated data: (1) Before the occurrence of BSI and RESP: to place high risk patients under more rigorous infection surveillance. (2) After the occurrence of BSI or RESP: to investigate “unexpected” infections. (3) At discharge: to identify high risk patients with no infections for further studies.

**Disclosures:**

**All Authors**: No reported disclosures

